# Supplementary material for: A NPAS4–NuA4 complex couples synaptic activity to DNA repair
Source: Nature. 2023 Feb 15;614(7949):732–41. doi: 10.1038/s41586-023-05711-7 (PMC9946837; doi:10.1038/s41586-023-05711-7)
Supplement: Supplementary file 1 — Supplementary Fig. 1: full scan images for western blots in Fig. 1b and Extended Data Figs. 1a,d–f,h–j, 2a,b,d and 4d. Supplementary Fig. 2: gating strategy for Fig. 5d and Extended Data Figs. 11d,e and 13a. [file 41586_2023_5711_MOESM1_ESM.pdf]

nature portfolio

https://doi.org/10.1038/s41586-023-05711-7

Supplementary information

A NPAS4–NuA4 complex couples synaptic activity to DNA repair

In the format provided by the authors and unedited

nature portfolio

https://doi.org/10.1038/s41586-023-05711-7

Supplementary information

A NPAS4–NuA4 complex couples synaptic activity to DNA repair

In the format provided by the authors and unedited

Supplementary Figure 1

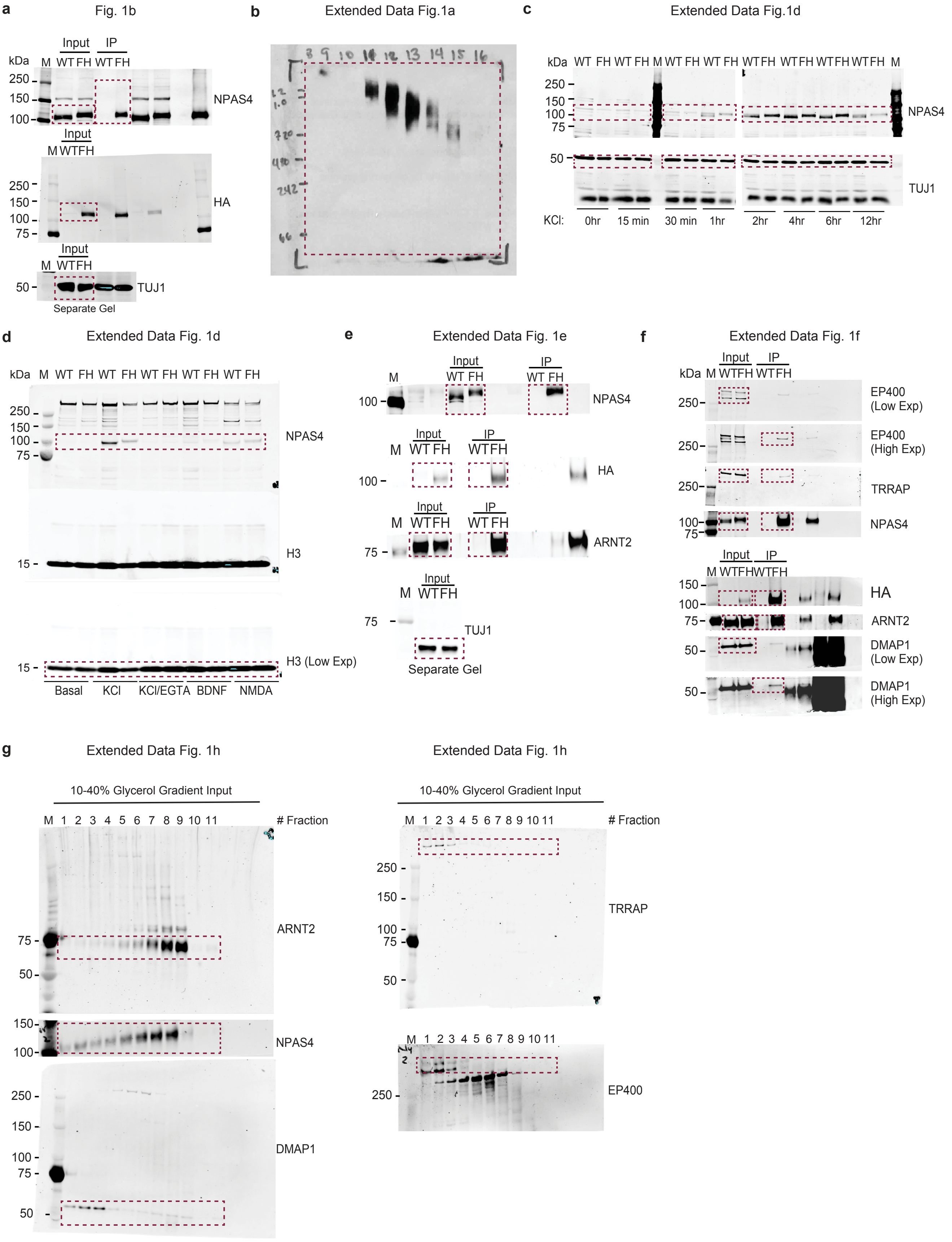

h Extended Data Fig. 1i

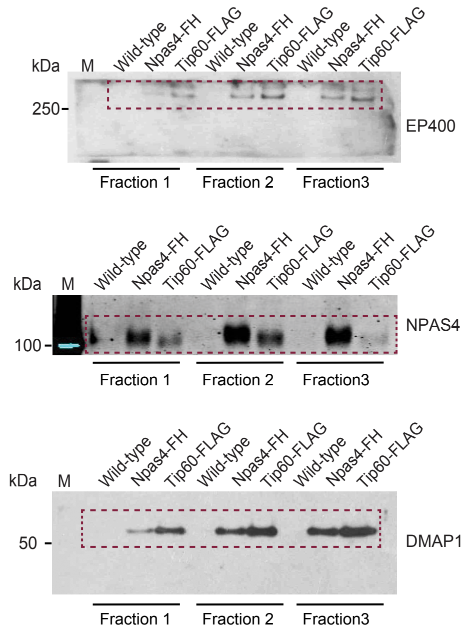

i Extended Data Fig. 1j

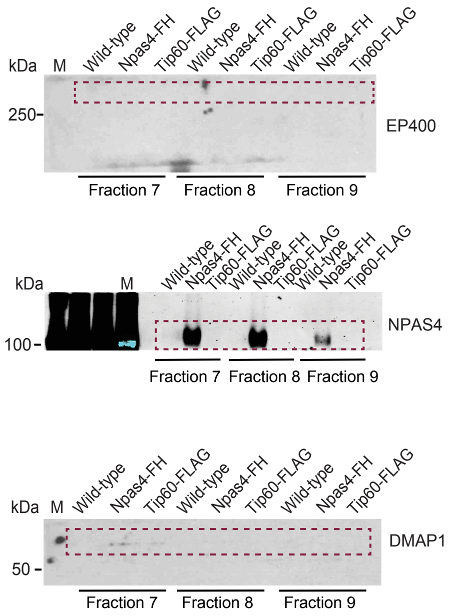

j Extended Data Fig. 2a

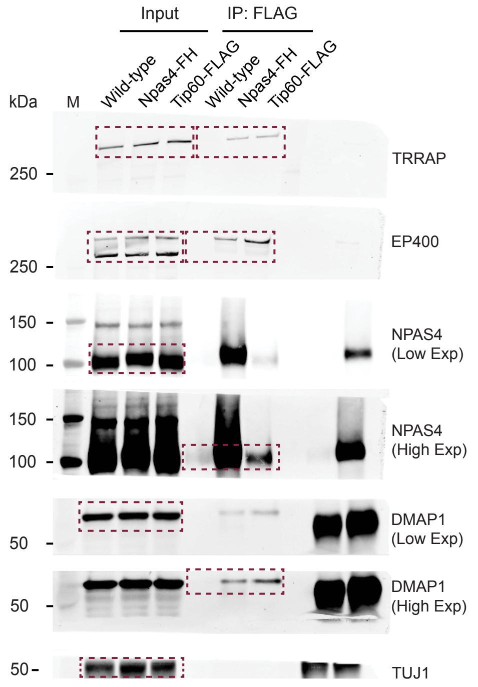

k Extended Data Fig. 2b

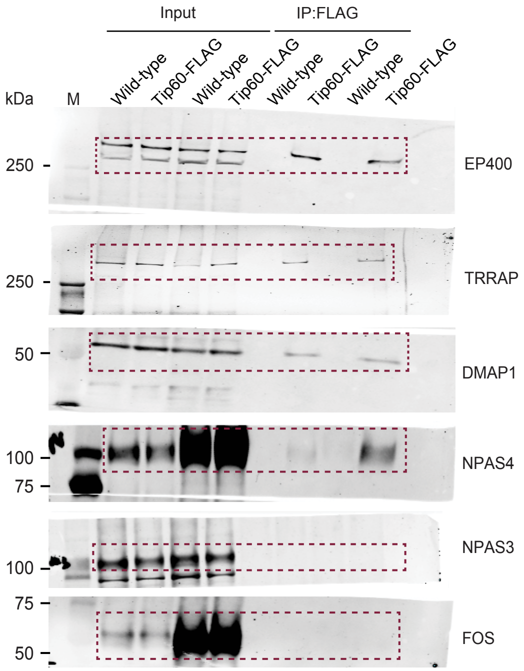

l Extended Data Fig. 2d Input — Extended Fig. 2d IP

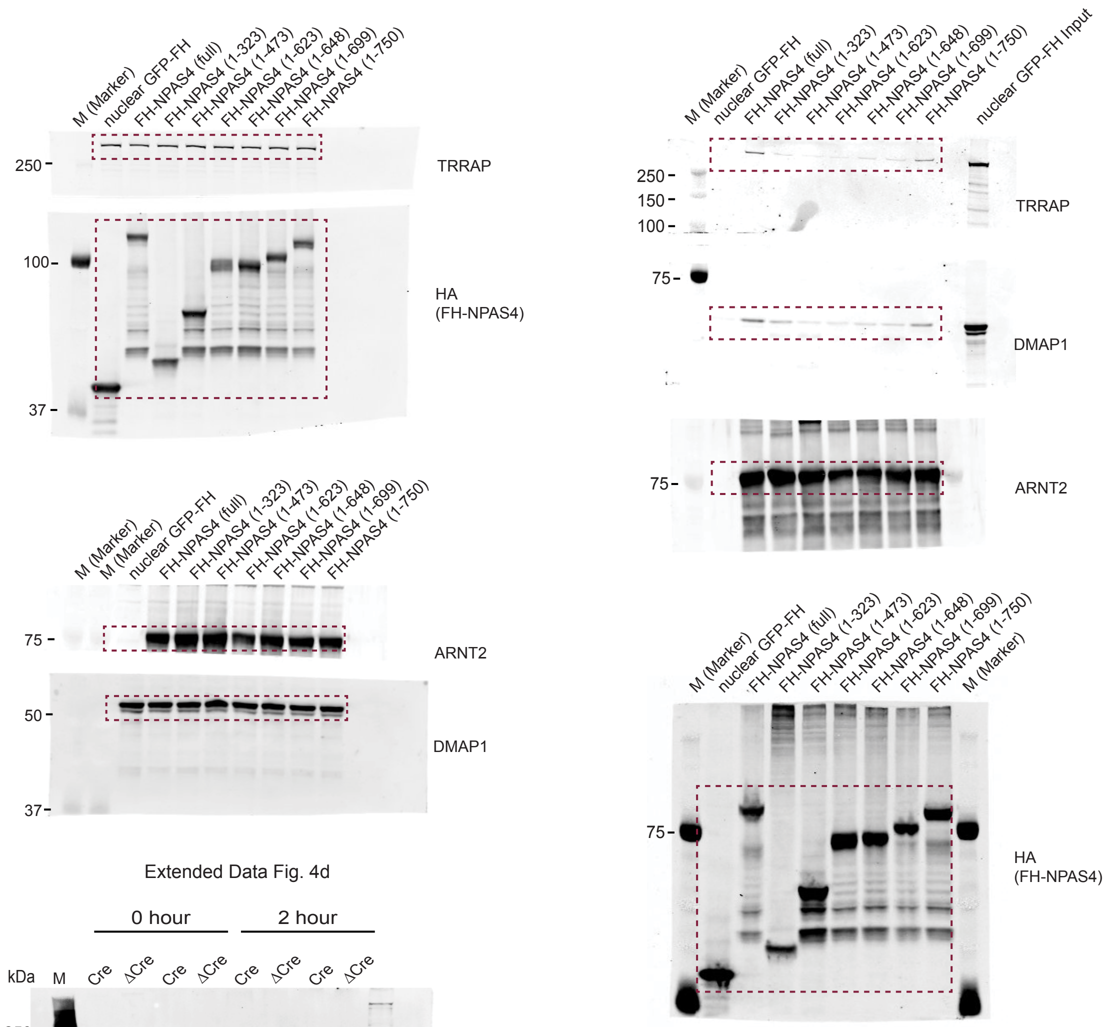

m Extended Data Fig. 4d

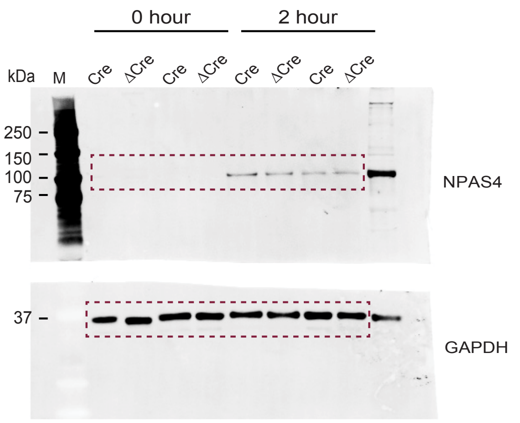

a-m) Uncropped western blots shown throughout manuscript. M=marker; red box indicates area cropped for the Figure. Separate gel indicates samples for which the processing control was run on a separate gel.

Supplementary Figure 2

a FACS gating strategy for sorting of Cre-mCherry- and ΔCre-GFP- infected hippocampal tissue

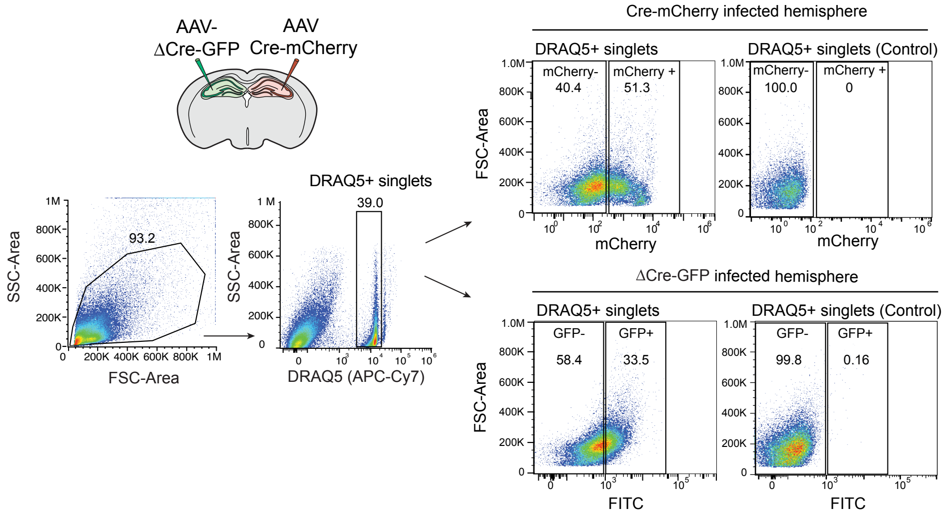

b FACS gating strategy for sorting NeuN+ nuclei from wild-type mice

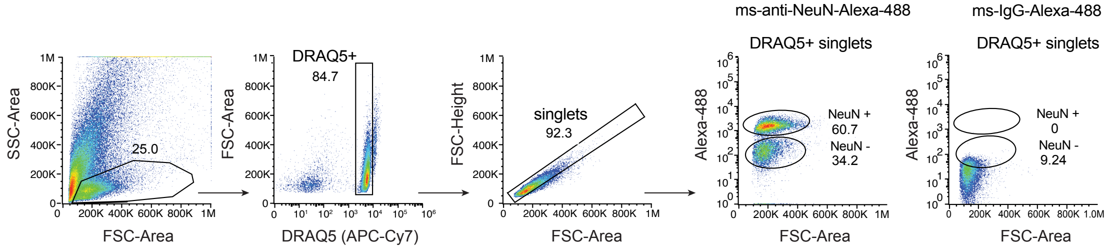

a) FACS gating of Cre-mCherry- and ΔCre-GFP-infected nuclei from hippocampal tissue. Unstained sample without DRAQ5 nuclear dye was used to establish the APC-Cy7-positive gate for DRAQ5-positive nuclei. Nuclei stained with DRAQ5 were initially selected based on APC-Cy7 signal, followed by selection of nuclei with proportional APC-Cy7 area and SSC signal to isolate singlet nuclei. mCherry-positive and GFP-positive gates were determined using a DRAQ5-positive sample from an uninfected mouse. **b)** FACS Gating of NeuN-positive neuronal nuclei from wild-type mice. Unstained sample without DRAQ5 nuclear dye was used to establish the APC-Cy7-positive gate for DRAQ5-positive nuclei. Nuclei stained with DRAQ5 were initially selected based on APC-Cy7 signal, followed by selection of nuclei with linearly proportional FSC area and height signal to isolate singlet nuclei. NeuN-positive gate was determined using both a DRAQ5-stained sample that was not stained with mouse anti-NeuN-Alexa488 (no primary control) and a DRAQ5-stained sample that was stained with mouse IgG-Alexa488 (isotype control).
