# Supplementary material for: A NPAS4–NuA4 complex couples synaptic activity to DNA repair
Source: Nature. 2023 Feb 15;614(7949):732–41. doi: 10.1038/s41586-023-05711-7 (PMC9946837; doi:10.1038/s41586-023-05711-7)
Supplement: Supplementary file 2 — Reporting Summary [file 41586_2023_5711_MOESM2_ESM.pdf]

nature portfolio

Corresponding author(s): Michael Greenberg

Last updated by author(s): Dec 12, 2022

Reporting Summary

Nature Portfolio wishes to improve the reproducibility of the work that we publish. This form provides structure for consistency and transparency in reporting. For further information on Nature Portfolio policies, see our Editorial Policies and the Editorial Policy Checklist.

Statistics

For all statistical analyses, confirm that the following items are present in the figure legend, table legend, main text, or Methods section.

n/a	Confirmed	
☐	☒	The exact sample size (*n*) for each experimental group/condition, given as a discrete number and unit of measurement
☐	☒	A statement on whether measurements were taken from distinct samples or whether the same sample was measured repeatedly
☐	☒	The statistical test(s) used AND whether they are one- or two-sided<br>*Only common tests should be described solely by name; describe more complex techniques in the Methods section.*
☒	☐	A description of all covariates tested
☐	☒	A description of any assumptions or corrections, such as tests of normality and adjustment for multiple comparisons
☐	☒	A full description of the statistical parameters including central tendency (e.g. means) or other basic estimates (e.g. regression coefficient) AND variation (e.g. standard deviation) or associated estimates of uncertainty (e.g. confidence intervals)
☐	☒	For null hypothesis testing, the test statistic (e.g. *F*, *t*, *r*) with confidence intervals, effect sizes, degrees of freedom and *P* value noted<br>*Give P values as exact values whenever suitable.*
☒	☐	For Bayesian analysis, information on the choice of priors and Markov chain Monte Carlo settings
☒	☐	For hierarchical and complex designs, identification of the appropriate level for tests and full reporting of outcomes
☐	☒	Estimates of effect sizes (e.g. Cohen's *d*, Pearson's *r*), indicating how they were calculated

Our web collection on statistics for biologists contains articles on many of the points above.

Software and code

Policy information about availability of computer code

Data collection	VS ASW-FL (Image acquisition software for VS120 Slide Scanner Microscope). FlowJo (10.0.8r1). Sony SH800Z FACS acquisition software. Illumina Next-Seq Control Software (v4.0.2)
Data analysis	Homer(v4.9), Prism (v8.4.2), R(3.6.1), EdgeR(3.28.1), Limma(3.42.2), Trimmomatic (0.36), vbowtie2(2.2.9), sBLISS-seq mapping pipeline (https://github.com/BiCroLab/blissNP.git), CellRanger(3.0.0), Seurat(v3), Monocle3, MACS2 (v 2.1.1), SEACR_1.1, bedtools (2.27.1), DESeq2 (1.26.0), Subread(v1.5.1), Debarcer_v0.3.1 (https://github.com/oicr-gsi/debarcer/releases/tag/v0.3.1), UCSC Genome Browser LiftOver (https://genome.ucsc.edu/cgi-bin/hgLiftOver), Meme-ChIP (https://meme-suite.org/meme/tools/meme-chip), Sequest (Thermo Fisher Scientific, Waltham, MA)

For manuscripts utilizing custom algorithms or software that are central to the research but not yet described in published literature, software must be made available to editors and reviewers. We strongly encourage code deposition in a community repository (e.g. GitHub). See the Nature Portfolio guidelines for submitting code & software for further information.

Data

Policy information about availability of data

All manuscripts must include a data availability statement. This statement should provide the following information, where applicable:

- Accession codes, unique identifiers, or web links for publicly available datasets
- A description of any restrictions on data availability
- For clinical datasets or third party data, please ensure that the statement adheres to our policy

All sequencing data for RNA-seq, ATAC-seq, ChIP-seq, CUT&RUN, sBLISS-seq, END-seq, snRNA-seq, and amplicon sequencing has been deposited in the Gene Expression Omnibus with accession number GSE175965. Mass spectrometry data has been deposited in PRIDE repository with accession number PXD038718. Raw gel images are provided in Supplementary Information Fig. 1. Additional data is provided as source data throughout the manuscript.

Human research participants

Policy information about studies involving human research participants and Sex and Gender in Research.

Reporting on sex and gender	N/A
Population characteristics	N/A
Recruitment	N/A
Ethics oversight	N/A

Note that full information on the approval of the study protocol must also be provided in the manuscript.

Field-specific reporting

Please select the one below that is the best fit for your research. If you are not sure, read the appropriate sections before making your selection.

☒ Life sciences ☐ Behavioural & social sciences ☐ Ecological, evolutionary & environmental sciences

For a reference copy of the document with all sections, see nature.com/documents/nr-reporting-summary-flat.pdf

Life sciences study design

All studies must disclose on these points even when the disclosure is negative.

Sample size	No statistical methods were used to predetermine sample size. Sample sizes were determined according to standards of practice in the field for each assay and generally adhere to guidelines of the ENCODE consortium (https://www.encodeproject.org/about/experiment-guidelines/). Sample size details are included in the manuscript with experimental description.
Data exclusions	For mutational analysis experiments, to include a given amplicon in our analysis, we required that the amplicon be found in greater than 1/3 of all samples and that the average number of consensus 10 families across all bases in the amplicons was >100. For information on primer pooling and the amplicons included in the final analysis, see Supplementary Table 5. For mutational analysis, extreme outlier points were removed across all conditions using a ROUT's test at 0.1% confidence performed in Prismv8.4.2.
Replication	Replicate details for each assay are provided in the manuscript. All attempts at replication were successful. For specific replicate numbers per assay, please see Supplementary Table 2. In general, in vitro RNA-seq experiments were performed in triplicate. RNA-seq from hippocampal tissue paired with sBLISS-seq data was performed with 8-10 replicates per timepoint. Single-nucleus RNA-seq experiments were performed on 2 independent Npas4fl/fl (Cre vs ΔCre) animals and 3 independent Tip60fl/fl (Cre vs ΔCre) animals. γH2AX ChIP-seq was performed in triplicate. ATAC-seq was performed in triplicate. All CUT&RUN experiments were conducted at least twice and for most antibodies in triplicate, with the exception of one EP400 dataset generated using an antibody from Bethyl Laboratories. This additional dataset corroborates data using a second EP400 antibody from Abcam, which has been conducted in duplicate in wild-type tissue and in triplicate in ΔCre (Control) infected animals. NPAS4 CUT&RUN was performed 5 times in wild-type mice and in duplicate in NPAS4 KO mice. IP-MS experiments on hippocampal tissue were performed in triplicate for the initial isolation of the complex. IP-MS experiments conducted from fractionated lysates were performed in duplicate. BLISS-seq experiments were conducted using 8-10 replicates in wild-type mice and three to five times in our Npas4 and Tip60 cKO (Cre vs ΔCre).
Randomization	All our data are derived from mice or cultured HEK293T cells. In general samples were grouped according to mouse age and genotype. Care was taken to include mice of both sexes equally in experimental groups.
Blinding	For genomic assays (ATAC-seq, RNA-seq, ChIP-seq, CUT&RUN, amplicon sequencing for mutational analysis, BLISS-seq, END-seq) and biochemistry, blinding of animal/tissue genotype was not feasible due to constraints in sample processing. All genomic assays are treated to the same bioinformatic pipelines. For electrophysiology experiments, the experimenter was blinded to animal genotype and treatment.

condition.

Reporting for specific materials, systems and methods

We require information from authors about some types of materials, experimental systems and methods used in many studies. Here, indicate whether each material, system or method listed is relevant to your study. If you are not sure if a list item applies to your research, read the appropriate section before selecting a response.

Materials & experimental systems

n/a	Involved in the study
☐	☒ Antibodies
☐	☒ Eukaryotic cell lines
☒	☐ Palaeontology and archaeology
☐	☒ Animals and other organisms
☒	☐ Clinical data
☒	☐ Dual use research of concern

Methods

n/a	Involved in the study
☐	☒ ChIP-seq
☐	☒ Flow cytometry
☒	☐ MRI-based neuroimaging

Antibodies

Antibodies used

HA (Cell Signaling Technology, C29F4, RRID AB_1549585), HA (Sigma-Aldrich, ROAHAHA); rabbit anti-NPAS4 (in house (Lin et al., Nature 2008); rabbit anti-ARNT2 (in house, Sharma et al., Neuron 2019); rabbit anti-KAT5 (Proteintech, 10827-1-AP, RRID AB_2128431); rabbit anti-Cleaved Caspase-3 (Cell Signaling Technology, 9664S, RRID AB_2070042); rabbit anti-EP400 (Bethyl Labs, A300-541-A, RRID AB_2098208); rabbit anti-EP400 (Abcam, Ab5201, RRID AB_304780), rabbit anti-EP400 (Abcam, 70301, RRID AB_1269644); rabbit anti-DMAP1 (Cell Signaling Technology, 13326, RRID AB_2798180); mouse anti-DMAP1 (Santa Cruz, sc373949, RRID AB_10918457); rabbit anti-TRRAP (Bethyl, A301-132A, RRID AB_2209668); rabbit anti-cFOS (in house); mouse anti-β-TUBULIN3 (Covance, MMS-435P, RRID AB_2313773), rabbit anti-GAPDH (Sigma-Aldrich, G9545, RRID AB_796208); rabbit anti-NPAS3 (Gift of Steven McKnight; Erbel-Sieler et al., PNAS 2004); rabbit anti-H3K27ac (Abcam, ab4729, RRID AB_2118291); rabbit anti-MRE11 (Novus Biologicals, Novus NB100-142, RRID AB_10077796, Lot R and S); rabbit anti-ETL4 (Bethyl Laboratories, A304-928A, RRID AB_2621122); anti-M2-FLAG 1224 resin (Sigma-Aldrich, A2220, RRID AB_10063035); Mouse anti-NeuN-Alexa488 (Millipore, MAB377X, clone A60, RRID AB_2149209); Mouse IgG-Alexa488 (Life Technologies, MA518167, RRID AB_2539541); rabbit anti-γH2AX (Abcam, ab2893, RRID AB_303388); rabbit anti-RAD50 (Novus Biologicals, NB100-154, RRID AB_2177080); rabbit anti-CTCF (Active Motif, 61311, RRID AB_2614975). For validation, see manufacturer's details for specificity using RRID from antibody registry.

Validation

Antibody validation was tailored to application. For Western blots, the following antibodies were used: rabbit anti-EP400 (Bethyl Labs, A300-541-A; Abcam, Ab5201; Abcam, 70301; 1:1000), rabbit anti-DMAP1 (Cell Signaling Technology, 13326; 1:500 or 1:1000) mouse anti-DMAP1 (Santa Cruz, sc373949; 1:500 or 1:1000), rabbit anti-TRRAP (Bethyl, A301-132A; 1:500 or 1:1000), rabbit anti-ARNT2 (in house; 1:1000), rabbit anti-NPAS4 (in house; 1:1000), rabbit anti-FOS (in house; 1:1000), mouse anti-β-TUBULIN3 (Covance, MMS-435P, 1:5000), rabbit anti-GAPDH (Sigma-Aldrich, G9545; 1:5000), rabbit anti-Histone H3 (Abcam 1791, 1: 10,000), rabbit anti-NPAS3 (Gift of Steven McKnight; 1:1000), rabbit anti-HA (Cell Signaling Technology, C29F4; 1:1000). Knockout or knockdown controls were performed to validate NPAS4, EP400, DMAP1, ARNT2, MRE11. HA validation was performed by blotting in wild-type mice lacking a transgenic epitope. Rabbit anti-NPAS4 was previously validated (Lin et al., Nature 2008). Rabbit anti-ARNT2 was previously validated (Sharma et al., Neuron, 2019). Rabbit anti-FOS in house antibody was previously validated (Yap et al., Nature 2021). For CUT&RUN, the following antibodies were used: rabbit anti-FOS (in house), rabbit anti-H3K27ac (Abcam, ab4729), rabbit anti-NPAS4 (in house), rabbit anti-EP400 (Bethyl Laboratories, A300541A; Abcam ab5201), rabbit anti-ARNT2 (in house), rabbit anti-MRE11 (Novus Biologicals, Novus NB100-142), rabbit anti-RAD50 (Novus Biologicals, NB100-154), rabbit anti-CTCF (Active Motif, 61311), rabbit anti-ETL4 (Bethyl Laboratories, A304-928A). For NPAS4 and MRE11, CUT&RUN signal was analyzed relative to knockout conditions. When KO conditions were difficult to obtain, multiple independent antibodies were used for a target protein (e.g. EP400). For immunohistochemistry, the following antibodies were used: rat anti-HA (Sigma-Aldrich, ROAHAHA, 1:250); rabbit anti-NPAS4 (in house, 1:1,000); rabbit anti-ARNT2 (in house, 1:1,000); rabbit anti-KAT5(TIP60) (Proteintech, 10827-1-AP, 1:250); rabbit anti-Cleaved Caspase-3 (Cell Signaling Technology, 9664S, 1:1,000). Antibody staining was validated in conditional knockout tissue or other negative controls (e.g. wild-type mice lacking a transgenic epitope). For FACS with staining, the following antibodies were used: mouse anti-NeuN-Alexa488 (Millipore, MAB377X, clone A60); Mouse IgG-Alexa488 (Life Technologies, MA518167). A non-targeting isotype control antibody was used as a negative control. NeuN staining and sorting was validated by qPCR for neuronal vs non-neuronal markers from sorted populations. For additional validation, see manufacturer's details for specificity using RRID from antibody registry listed above.

Eukaryotic cell lines

Policy information about cell lines and Sex and Gender in Research

Cell line source(s): HEK293T (Thermo Fisher Scientific # 50188404FP)

Authentication: Not tested

Mycoplasma contamination: Not tested

Commonly misidentified lines (See ICLAC register): No commonly misidentified cell lines were used in this study.

Animals and other research organisms

Policy information about studies involving animals; ARRIVE guidelines recommended for reporting animal research, and Sex and Gender in Research

Laboratory animals	Animal use was approved and overseen by Harvard University Institutional Animal Care and Use Committee and Harvard Center for Comparative Medicine. The following mouse lines were used: wild-type C57/BL6 (Jackson Labs Stock 000664), Npas4fl/fl (Lin et al., 2008), Npas4 KO (Lin et al., Nature 2008), Tip60fl/fl (Fisher et al., Plos One 2016), Npas4-FLAG-HA (this manuscript), Arnt2-FLAG-HA (this manuscript), Tip60-H3F (Chen et al., eLife 2013), Mre11fl/fl (Buis et al., Cell 2008, Nat Struct Mol Biol 2012), B6;129-Gt(ROSA)26Sor<tm5(CAG-Sun1/sfGFP)Nat>/J (Jackson labs Stock 021039), B6.Cg-Tg(Camk2a-cre)T29-1Stl/J (Jackson labs Stock 005329). Mice were housed in a temperature and humidity-controlled environment using ventilated micro-isolator cages. Mice were kept under a standard 12 hr light/dark cycle, with food and water provided ad libitum. Male and female littermate mice were used in similar proportions and divided between control and experimental groups for all experiments conducted. In the case of NPAS4 KO lifespan analysis, data is also separated by sex. See Extended Data 13. For biochemistry and genomic experiments, animals were collected at 4-6 weeks of age throughout the manuscript. For physiology experiments, animals were dissected and patched at P24-P28 . For aging experiments, animals were collected at 3-4 months, 12 months, and 23-27 months of age. Details of animal age and sex are detailed within each protocol.
Wild animals	Did not involve wild animals
Reporting on sex	Findings apply to mice of both sexes as male and female littermate mice were used in similar proportions and divided between control and experimental groups for all experiments conducted. In the case of the Npas4KO mice lifespan studies, we report individual results by sex.
Field-collected samples	No field samples collected
Ethics oversight	Animal use was approved overseen by Harvard University Institutional Animal Care and Use Committee and Harvard Center for Comparative Medicine.

Note that full information on the approval of the study protocol must also be provided in the manuscript.

ChIP-seq

Data deposition

☒ Confirm that both raw and final processed data have been deposited in a public database such as GEO.

☒ Confirm that you have deposited or provided access to graph files (e.g. BED files) for the called peaks.

Data access links<br>*May remain private before publication.*	Sequencing data have been deposited in the Gene Expression Omnibus with accession number GSE175965. Additional data are provided in source data files.
Files in database submission	Sequencing data have been deposited in the Gene Expression Omnibus with accession number GSE175965.
Genome browser session<br>(e.g. UCSC)	Graph files (bigWig) are included in Gene Expression Omnibus GSE175965.

Methodology

Replicates	Replicate information provided in Supplementary Table 2, Methods, and Figure Legends. Briefly, CUT&RUN experiments were conducted at least twice and for most antibodies in triplicate, with the exception of one EP400 dataset generated using an antibody from Bethyl Laboratories A300541A. This additional dataset corroborates data using a second EP400 antibody from Abcam, which has been conducted in duplicate in wild-type tissue and in triplicate in ΔCre (Control) infected animals. NPAS4 CUT&RUN was performed 5 times in wild-type mice and in duplicate in NPAS4 KO mice. γH2AX ChIP-seq was performed in triplicate.
Sequencing depth	Sequencing depth information provided in Supplementary Table 2. Briefly, all CUT&RUN experiments were sequenced on average with 22 million adapter trimmed pairs, with a minimum of 8 million adapter trimmed pairs. anti-γH2AX ChIP-seq samples were sequenced to a minimum depth of 20 million reads.
Antibodies	ChIP-seq: rabbit anti-γH2AX (Abcam, ab2893); rabbit anti-NPAS4 (in house); CUT&RUN: rabbit anti-NPAS4 (in house), rabbit anti-FOS (in house), rabbit anti-H3K27ac (Abcam, ab4729), rabbit anti-EP400 (Bethyl Laboratories, A300541A; Abcam ab5201), rabbit anti-ARNT2 (in house), rabbit anti-MRE11 (Novus Biologicals, NB100-142), rabbit anti-RAD50 (Novus Biologicals, NB100-154), rabbit anti-CTCF (Active Motif, 61311), rabbit anti-ETL4 (Bethyl Laboratories, A304-928A).
Peak calling parameters	Peak calling on ChIP samples was performed using MACS2 (macs2/2.1.1) using the following command macs2 callpeak –t (experimental bam) –c (input bam) -f BAM -g mm -p 1e-5. Required p less than 1e-5 in peak calling for ChIP-seq. All peak calling for CUT&RUN was performed using SEACR_1.1.sh (Meers et al., 2019). For H3K27ac, FOS, CTCF, and RAD50 CUT&RUN datasets, peak calling on individual replicates was performed using the spikein normalized bedgraph files based on fragments 1 to 1000 bp in length with the following command: SEACR_1.1.sh [target bedgraph] [control bedgraph] norm stringent. For NPAS4, ARNT2, EP400, MRE11, and ETL4 CUT&RUN datasets, peak calling on individual replicates was performed using SEACR_1.1.sh using the spikein normalized bedgraph files based on fragments 1 to 1000 bp in length with the following command: SEACR_1.1.sh [target bedgraph] [control bedgraph] norm relaxed. Paired control samples (either IgG or KO control) are listed in Supplementary Table 2.

Data quality	To identify reproducible peak sets for CUT&RUN, SEACR peaks found in 3 of 3 H3K27ac replicates (0 and 2hr KA stimulation), 3 of 3 FOS replicates (0 and 2hr KA stimulation), 3 of 3 CTCF replicates (0 and 2hr KA stimulation), 3 of 3 RAD50 replicates (0hr) and 3 of 4 RAD50 replicates (2hr KA stimulation), 4 of 5 NPAS4 replicates (2hr KA stimulation), 3 of 3 NPAS4 replicates (0hr KA stimulation), 2 of 2 ARNT2 replicates (2hr KA stimulation), 2 of 2 EP400 replicates (0 and 2hr KA stimulation; Abcam antibody), 2 of 2 MRE11 replicates (0 and 2hr KA stimulation), 2 of 2 ETL4 replicates (0hr KA stimulation) and 3 of 4 ETL4 replicates (2hr KA stimulation) were intersected using bedtools/2.27.1 intersect bed. Peaks within 150 bp were merged. Finally, the maxima of CUT&RUN signal within 100 bp windows for each peak was calculated from spikein normalized bigWig files using custom scripts. For FOS, ARNT2, EP400, ETL4, and MRE11, final peak calls were extended 200 base pairs up and downstream from this peak maxima to generate 500 base pair peak calls for each factor and timepoint. Mm10 blacklisted regions were filtered out using the following command: bedops/2.4.30 –not-element-of 1 [BLACKLIST_BED]. NPAS4 peaks were extended to 1 kb, as we found maximal enrichment for the Ebox (CAGATG) motif and bHLH/PAS motif (CGTG) in 1 kb regions extended from peak maxima.
Software	Peak calling on ChIP samples was performed using MACS2 (macs2/2.1.1). ChIP-seq samples were aligned to the mm10 genome using the Bowtie alignment software(vbowtie2/2.2.9) with the –very-sensitive setting. CUT&RUN peak calling was performed with SEACR_1.1.sh.

Flow Cytometry

Plots

Confirm that:

☒ The axis labels state the marker and fluorochrome used (e.g. CD4-FITC).

☒ The axis scales are clearly visible. Include numbers along axes only for bottom left plot of group (a 'group' is an analysis of identical markers).

☒ All plots are contour plots with outliers or pseudocolor plots.

☒ A numerical value for number of cells or percentage (with statistics) is provided.

Methodology

Sample preparation	For GFP and mCherry FACS: Dissected hippocampal tissue was examined under a florescent scope to detect GFP or mCherry. Tissue that was uninfected, or in rare cases showed infection of both fluorophores in a single hemisphere, was discarded. Hippocampi were placed in 0.5 mL of buffer HB (0.25 M sucrose, 25 mM KCl, 5 mM MgCl2, 20 mM Tricine-KOH, pH 7.8, 1 mM DTT, 0.15 mM spermine, 0.5 mM spermidine) and dounced 5X with a loose pestle and 10X with a tight pestle. 5% IGEPAL CA-630 (32 µL) was added prior to douncing with a tight pestle 5-8 more times and filtering through a 40-µm strainer. DRAQ5 nuclear dye (Abcam; ab108410) was added (1:500), and nuclei expressing either mCherry or GFP were sorted on a SONY SH800. Negative gates were determined using uninfected tissue. Nuclei were collected in 1 mL of CUT&RUN Wash Buffer containing 2 mM EDTA.<br>For NeuN-FACS: To sort neuronal nuclei for DNA isolation, dissected hippocampal tissue was placed in 1 mL of buffer HB (0.25 M sucrose, 25 mM KCl, 5 mM MgCl2, 20 mM Tricine-KOH, pH 7.8, 1 mM DTT, 0.15 mM spermine, 0.5 mM spermidine) and dounced 5X with a loose pestle and 10X with a tight pestle. 5% IGEPAL CA-630 (32 µL) was added prior to douncing again with a tight pestle 5-8 times. The nuclei suspension in HB was filtered through a 40-µm strainer. Nuclei were then pelleted by centrifugation at 500 g for 5 min and resuspended in 400 µL of FACS Block/Stain Buffer (1% BSA, 0.05% Igepal-630, 3 mM MgCl2 in 1X PBS). Nuclei were incubated for 15 min with gentle rotation at 4°C. Following this blocking step, nuclei were pelleted and resuspended in FACS Block/Stain Buffer containing 1:1000 dilution of mouse anti-NeuN-Alexa488 (Millipore, MAB377X). An isotype control Mouse IgG-Alexa488 (Life Technologies, MA518167) was included as a negative control along with an unstained sample. Samples were incubated in antibody mix for 1 hour with gentle rotation at 4°C. Nuclei were washed 1X with FACS Block/Stain Buffer, and DRAQ5 nuclear dye (Invitrogen) was added (1:500) prior to sorting. NeuN-high-expressing nuclei were separated from NeuN-low-expressing nuclei using a SONY SH800.
Instrument	SONY SH800
Software	Sony SH800Z Cell Sorter software was used during acquisition of data. Data were subsequently analyzed using FlowJo (10.0.8r1)
Cell population abundance	Singlet DRAQ5-positive nuclei represented roughly 25% of the initial population. NeuN+ nuclei gated from the singlet DRAQ5-positive population represented roughly 60% of the population. Cre-mCherry+ and ΔCre-GFP+ nuclei abundance depended on the viral injection and tissue microdissection and ranged from 20% to 75% of the singlet DRAQ5-positive population. See Extended Data Figs. 11d,e and 13a and Supplementary Fig. 2 for gating strategy.

Gating strategy

For NeuN-FACS: Unstained sample without DRAQ5 nuclear dye was used to establish the APC-Cy7-positive gate for DRAQ5-positive nuclei. Nuclei stained with DRAQ5 were initially selected based on APC-Cy7 signal, followed by selection of nuclei with linearly proportional FSC area and height signal to isolate singlet nuclei. NeuN+ gate was determined using both a DRAQ5-stained sample that was not stained with Mouse anti-NeuN-Alexa488 (no primary control) and a DRAQ5-stained sample that was stained with a Mouse IgG-Alexa488 isotype control. See Extended Data 13a and Supplementary Fig. 2 for gating strategy.
For mCherry and GFP FACS: Unstained sample without DRAQ5 nuclear dye was used to establish the APC-Cy7-positive gate for DRAQ5-positive nuclei. Nuclei stained with DRAQ5 were initially selected based on APC-Cy7 signal, followed by selection of nuclei with proportional APC-Cy7 area and SSC signal to isolate singlet nuclei. mCherry+ and GFP+ gates were determined using a DRAQ5-positive sample from an uninfected mouse. See Extended Data 11d,e and Supplementary Fig. 2 for gating strategy.

☒ Tick this box to confirm that a figure exemplifying the gating strategy is provided in the Supplementary Information.
